# Mismatch Repair Protein Loss as a Prognostic and Predictive Biomarker in Breast Cancers Regardless of Microsatellite Instability

**DOI:** 10.1093/jncics/pky056

**Published:** 2018-12-13

**Authors:** Nicola Fusco, Gianluca Lopez, Chiara Corti, Chiara Pesenti, Patrizia Colapietro, Giulia Ercoli, Gabriella Gaudioso, Alice Faversani, Donatella Gambini, Anna Michelotti, Luca Despini, Concetta Blundo, Valentina Vaira, Monica Miozzo, Stefano Ferrero, Silvano Bosari

**Affiliations:** 1 Division of Pathology, Fondazione IRCCS Ca’ Granda, Ospedale Maggiore Policlinico, Milan, Italy; 2 Department of Biomedical, Surgical, and Dental Sciences; 3 Department of Pathophysiology & Transplantation, Università degli Studi di Milano, Milan, Italy; 4 Division of Medical Oncology (DG, AM); 5 Division of Breast Surgery, Fondazione IRCCS Ca’ Granda - Ospedale Maggiore Policlinico, Milan, Italy

## Abstract

**Background:**

Breast cancers that harbor mismatch-repair (MMR) deficiency and/or microsatellite instability (MSI) might be sensitive to immune checkpoint blockade, but there are currently no specific guidelines for assessing MMR status in breast cancer. Here, we sought to define the clinical value of MMR immunohistochemistry (IHC) and MSI analysis in breast cancers.

**Methods:**

We subjected 444 breast cancers to MMR IHC and MSI analysis. Cases were classified as MMR-proficient (pMMR), MMR-deficient (dMMR), and MMR-heterogeneous (hMMR) based on the loss of immunoreactivity; MSI was defined by instability in the five indicators recommended by the National Cancer Institute for endometrial and colorectal cancers. Correlation of MMR status with patients’ survival was assessed using the Kaplan-Meier estimator. Statistical tests were two-sided.

**Results:**

Loss of MMR proteins was homogeneous (dMMR) in 75 patients (17%) and heterogeneous (hMMR) in 55 (12%). Among luminal breast cancers, there were similar frequencies of dMMR and hMMR tumors. Overall, the rate of discrepancy between IHC and MSI analysis was high (91%). Women with Luminal B-like dMMR carcinomas (n = 44) showed shorter overall survival (median = 77 months, range = 0–115 months) than those with pMMR (n = 205) or hMMR (n = 35) tumors (median = 84 months, range = 0–127 months) (*P* = .008). On the contrary, patients with estrogen receptor-negative breast cancers treated with chemotherapy lived longer in cases of dMMR (n = 9) than pMMR (n = 33) or hMMR (n = 7) tumors, with 87 months of median survival (range = 73–123 months) for the former compared with 79 months (range = 8–113 months) for the latter two categories (*P* < .001).

**Conclusions:**

Immunohistochemistry and MSI are not interchangeable tests in breast carcinomas. MMR protein loss is a more common event than MSI and shows intra-tumor heterogeneity. MMR IHC allows the identification of clinically relevant subclasses of breast cancer patients, provided that multiple areas of the tumor are analyzed.

Mismatch repair (MMR) is a crucial biological system for the recognition and correction of base mispairs generated during DNA replication and recombination (1). Four proteins are the main components of this complex: mutL homolog 1 (MLH1), mutS homolog 2 (MSH2), mutS homolog 6 (MSH6), and postmeiotic segregation increased 2 (PMS2) (1,2). Mutations in *MLH1*, *MSH2*, *MSH6*, and *PMS2* and/or the epigenetic silencing of *MLH1* or *MSH2* genes can trigger MMR malfunction, which induces genome instability and promotes cancer (2,3). Recognition of MMR-deficient (dMMR) neoplasms is becoming more and more important (2,4–6). In 2017, the US Food and Drug Administration approved second-line pembrolizumab —a drug targeting the immune-checkpoint programmed cell death protein 1— in any tumor, thus including breast cancer, showing MMR deficiency and/or high levels of microsatellite instability (MSI-H) (7). This is the first time that an international public health agency has approved a cancer treatment based on biomarkers rather than tumor site or histology (8). Regrettably, the identification of patients with dMMR cancers is currently based on vastly heterogeneous, locally developed laboratory tests.

Although MMR alterations are driver events in a subset of breast cancers (6,9), their identification is not routine in breast pathology. Clinical and histopathologic criteria associated with immunohistochemistry (IHC), polymerase chain reaction-based testing of microsatellite loci, and sequencing methylation assays are common practice in the diagnosis of MMR deficiency (3,10–12). These tools are also widely adopted in translational research studies for identifying dMMR breast cancers (6,9,13). It should be noted, however, that the analytical criteria for MMR IHC and microsatellite instability (MSI) have been adapted from those tumors where MMR defects are frequently inherited, such as colorectal and endometrial cancers (5,10,14,15). To date, there are no validated methods for the assessment of MMR status in breast cancers. Hence, the overall response rate to pembrolizumab in unselected patients with advanced triple-negative (ie, estrogen receptor [ER]-, progesterone receptor [PR]-, and HER2-negative) breast cancers (TNBCs) was 18.5% in a multicenter, nonrandomized phase Ib clinical trial (16). Furthermore, even though they represent the vast majority of cases, only a few data on response to immune checkpoint blockade in ER+ breast cancers are available to date. In this scenario, the identification of subpopulations of breast cancer patients that could be more sensitive to immunotherapy is clinically relevant.

Our study aims to evaluate the clinical value of MMR testing in breast cancers. Here, we characterized the MMR status in a large series of breast cancers to define 1) the frequency and clinicopathologic features of dMMR breast cancers, 2) the concordance and interchangeability of IHC and MSI analysis for these patients, 3) the impact of intra-tumor heterogeneity, and 4) the prognostic and predictive role of MMR defects in breast cancers.

## Methods

### Patients and Tissue Specimens

A total of 444 breast cancers (2004–20^17^) were retrieved from the pathology archives of IRCCS Ca’ Granda Foundation – Policlinico Maggiore Hospital, Milan, Italy. Only patients diagnosed and managed in this institution whose tumors were greater than 5 mm in size and for which all histologic slides and blocks were available for review, as well as detailed clinical and follow-up data, were included. Patients with previous diagnosis of breast, gynecological, and colorectal malignancies, with a family history of breast cancer, fulfilling the Revised Bethesda Guidelines for the identification of individuals at risk for Lynch syndrome (including MLH1 IHC-negative MSI-H tumors showing no methylation of the *MLH1* promoter) (9,17), or who received neoadjuvant therapy were excluded. Samples were anonymized prior to analysis and the study was approved by the local institutional review board. All cases were reclassified and graded following the latest World Health Organization criteria (18) and the Nottingham grading system (19), respectively. Pathologic restaging was performed according to the eighth edition of the AJCC Cancer Staging Manual (20). Breast cancer intrinsic molecular subtypes were determined by ER, PR, Ki67, and HER2 status following the 2017 St Gallen International Expert Consensus recommendations (21). All clinicopathologic features and treatment information are summarized in Table 1

**Table 1.** Clinicopathologic features and treatment information of the patients included in the study

		Therapy				
Characteristic	No. of patients (%)	HT (%)	CT (%)	TTZ (%)	RT (%)	None (%)
All patients	444	259 (58)	190 (43)	13 (3)	300 (68)	8 (2)
Age at diagnosis, y						
≥55	311 (60)	204 (66)	104 (33)	10 (3)	221 (71)	7 (2)
<55	133 (30)	55 (41)	86 (65)	3 (2)	79 (59)	1 (1)
T status						
pT1	262 (59)	147 (56)	106 (41)	3 (1)	208 (79)	6 (2)
pT2	147 (33)	98 (67)	62 (42)	8 (5)	82 (56)	2 (1)
pT3–4	35 (8)	14 (40)	22 (63)	2 (6)	10 (29)	0
N status						
pN0	257 (58)	169 (66)	71 (28)	4 (2)	195 (76)	7 (3)
pN1–3	187 (42)	90 (48)	119 (64)	9 (5)	105 (56)	1 (1)
Histological grade						
1	46 (10)	26 (57)	12 (26)	0	38 (83)	3 (7)
2	190 (43)	130 (68)	56 (30)	1 (1)	132 (70)	3 (2)
3	208 (47)	103 (50)	122 (59)	12 (6)	130 (63)	2 (1)
Hormone receptor status						
Positive	392 (88)	259 (66)	145 (37)	9 (2)	271 (69)	4 (1)
Negative	53 (12)	0	45 (85)	4 (8)	29 (55)	4 (9)
HER2 status						
Positive	81 (18)	47 (58)	41 (51)	13 (16)	52 (64)	1 (1)
Negative	363 (82)	212 (58)	149 (41)	0	248 (68)	7 (2)
Ki67 status						
High	302 (68)	163 (54)	155 (51)	13 (4)	195 (65)	6 (2)
Low	142 (32)	96 (68)	35 (25)	0	105 (74)	2 (1)
Histological subtype						
Invasive carcinoma, NST	344 (77)	193 (56)	160 (47)	11 (3)	230 (67)	4 (1)
Lobular	53 (12)	39 (74)	13 (25)	1 (2)	40 (76)	1 (2)
Other	47 (11)	27 (58)	17 (36)	1 (2)	30 (64)	3 (6)
Intrinsic molecular subtype						
Luminal A-like*	108 (24)	76 (70)	28 (26)	0	81 (75)	1 (1)
Luminal B-like (HER2+)†	73 (16)	47 (64)	34 (47)	9 (12)	49 (67)	1 (1)
Luminal B-like (HER2-)‡	211 (48)	136 (65)	84 (40)	0	142 (67)	3 (1)
HER2-type§	8 (2)	0	7 (88)	4 (50)	3 (38)	0
TNBC‖	44 (10)	0	37 (84)	0	25 (57)	3 (7)

*ER+/PR+/Ki67 low. All cases were reclassified, regraded, and reassessed for hormone receptor, Ki67, and HER2 status according the latest guidelines (18–20). Treatment data refer to the therapy performed using the different clinical protocols and guidelines during the follow-up period (2004–2017). NST = no special type; HT = hormone therapy; CT = chemotherapy; TTZ = trastuzumab; RT = radiotherapy; TNBC = triple-negative breast cancer.

†ER+/PR+/HER2+/Ki67 high or ER+/PR−/HER2+.

‡ER+/PR+/HER2−/Ki67 high or ER+/PR−/HER2−.

§ER−/PR−/HER2+.

‖ER−/PR−/HER2−.

.

### Tissue Microarrays (TMAs) Construction

For each case, samples from the tumor core, invasive front, in situ component, and non-neoplastic glandular breast tissue were incorporated into 15 TMAs optimized for the high-throughput analysis of different topographic areas in large cohorts of heterogeneously processed breast tumors, as previously described (22). Each TMA block contained up to 180 tumor cores measuring 2 mm in diameter, with a total number of 2790 spots of tissue (mean of 5.8 tumor tissue cores per case, range = 2–7 cores).

### Immunohistochemical Analysis

Representative 4-μm-thick sections were cut from the TMA blocks and subjected to IHC using prediluted antibodies against ER, PR, Ki67, HER2, MLH1, MSH2, MSH6, and PMS2. Positive and negative controls were included in each slide run. Briefly, the protocols use two automated staining systems (ie, Dako Omnis and Ventana Benchmark Ultra) and anti-human prediluted antibodies (23). Protein expression was analyzed separately in all morphologically different tumor components by two independent pathologists (NF and GL) (24). Discordant results were resolved during dedicated consensus sessions. The methods and scoring systems employed followed previously reported criteria and guidelines (10,25–28), as detailed in Supplementary Table 1 (available online). Specifically, loss of MMR protein expression was defined by the complete absence of nuclear staining within all neoplastic cells (15). Cancers showing retained protein expression of the four MMR proteins across the entire tumor were defined as MMR-proficient (pMMR), and the diffuse loss of one or more MMR proteins designated the dMMR status. When the protein was expressed in only a part of the tumor (ie, <50% of tumor cells within ≥1 tissue core, as shown in Supplementary Figure S1, available online), the case was recorded as MMR-heterogeneous (hMMR). Whole tissue tumor sections from all dMMR and 25 pMMR cases were subjected to MMR IHC to confirm their non-hMMR phenotype.

### Silver in Situ Hybridization

Three-μm-thick sections from all TMAs underwent silver in situ hybridization using probes for *HER2* and its corresponding centromere (CEP17) and were assessed according to the American Society of Clinical Oncology (ASCO)/College of American Pathologists (CAP) recommendations (26), as detailed elsewhere (22).

### Cancer Cell Enrichment and DNA Extraction

Tumor and matched normal breast tissues of all hMMR and dMMR cases were manually microdissected using a sterile needle under a stereomicroscope (Stemi 305, Zeiss, Germany) to ensure more than 85% of tumor cell content and that the normal tissue was devoid of atypical cells (29). Microdissection was performed by two of the authors (GL and CC) under the supervision of a breast pathologist (NF). Genomic DNA extraction was performed as described (30).

### Microsatellite Instability Analysis

All dMMR breast cancers and matched normal tissues were subjected to MSI testing using the five indicators recommended by the National Cancer Institute (31), which included two quasimonomorphic mononucleotides (BAT25 and BAT26) and three polymorphic dinucleotides (D2S123, D5S346, and D17S250). The polymerase chain reaction primer sequences are listed in Supplementary Table 2 (available online). The analysis was performed by capillary gel electrophoresis using the Gene Mapper software on an ABI 3130XL system (Applied Biosystems, Foster City, CA). Tumors were considered MSI-positive when alterations appeared in the microsatellite profiles of the tumor sample (ie, mismatch in >1 major electropherogram peaks compared to the non-neoplastic counterpart) (31). Low-frequency MSI and MSI-H were defined by the presence of one and two or more unstable markers, respectively (5,10,28).

### Statistical Analyses

Differences in MMR proteins expression across tumor types were investigated using the χ^2^ test (IBM SPSS). To assess the correlation between the clinicopathologic features and MMR protein loss, nonparametric models were applied first using the MMR status of each case in a dual fashion (pMMR/dMMR). A second proportional-hazards regression analysis taking into account the hMMR status was subsequently performed. Correlation of the protein’s patterns of expression to patients’ overall and disease-free survival was assessed using the Kaplan-Meier estimator. Patients who died from causes other than breast cancer (n = 10) were labeled as censored at death for survival analysis. Quantitative analyses were performed using the multiple Cox proportional hazards regression to assess the independence of MMR IHC as a prognostic factor (32). Two-sided probability values (*P*) less than .05 were considered statistically significant.

## Results

### Mismatch Repair Protein Expression and Intra-Tumor Heterogeneity

**Figure 1.**
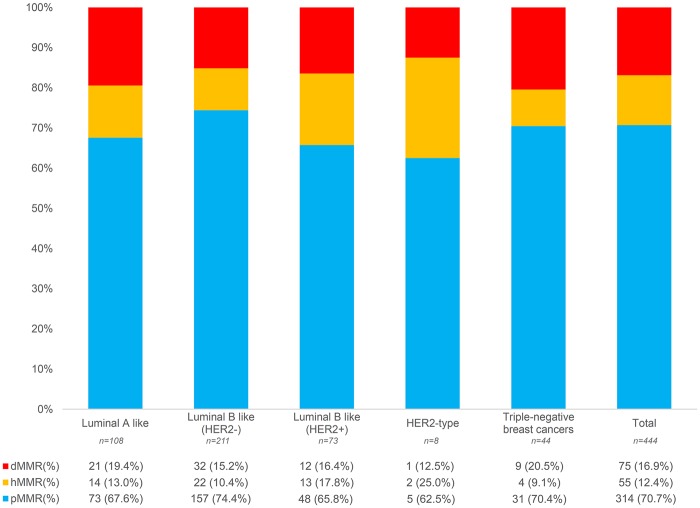
Mismatch repair status of 444 sporadic breast cancers according to the intrinsic molecular subtypes. Bar graph showing the uniform relative proportion of mismatch repair deficient (dMMR), heterogeneous (hMMR), and proficient (pMMR) breast cancers across the intrinsic molecular subtypes. The three immunohistochemical scenarios are color-coded on the basis of the legend on the bottom left.

Among the 444 breast cancers analyzed, 41 (9%), 55 (12%), 24 (10%), and 21 (5%) cases showed homogeneous loss of MLH1, MSH2, MSH6, and PMS2, respectively, and the cases showing intra-tumor heterogeneity ranged from 7 (1%) for PMS2 to 42 (10%) for MSH2 (Supplementary Table 3, available online). The relative frequency of heterogeneous protein expression ranged from 33% for PMS2 to 50% for MSH6, with higher rates in Luminal cancers, as depicted by the black bars in Supplementary Table 3 (available online). Taken together, 314 (71%) breast cancers were pMMR and 75 (17%) and 55 (12%) tumors were dMMR and hMMR, respectively (Figure 1 ). The prevalence of carcinomas showing loss of MMR proteins across the intrinsic molecular subtypes ranged from 15% to 21% for dMMR (mean 16.8%) and 9% to 25% for hMMR (mean 15.1%), as shown in Figure 1 and Supplementary Tables 3 and 4 (available online). No hMMR tumors were observed in pT3–4 Luminal A-like and Luminal B-like (HER2+) tumors or in pT2–4 TNBCs. Among the other pathologic (p)T and pN categories, the prevalence of hMMR tumors ranged from 7% to 50% (Supplementary Figure 2, available online). These findings suggest that the MMR protein analysis in a single area of the tumor may not represent the MMR status of the entire breast neoplasm.

### Microsatellite Instability in Breast Cancers with MMR Protein Loss

**Figure 2.**
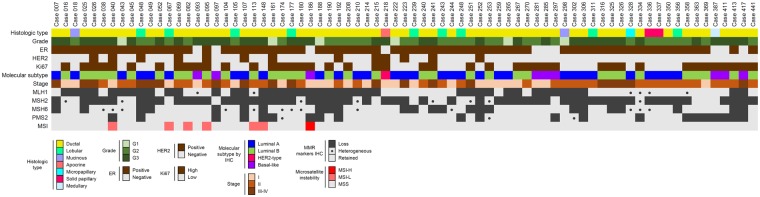
Overview of 75 mismatch repair deficient breast carcinomas. Heatmap illustrating the clinical, histologic, and biological information together with mismatch repair protein status and microsatellite instability data of all mismatch repair-deficient cases identified. Each column represents a case, each row a parameter, which is color-coded according to the legend below. ER = estrogen receptor; IHC = immunohistochemistry; MSI = microsatellite instability; MSI-H = microsatellite instability high; MSI-L = microsatellite instability low.

All 75 dMMR tumors were analyzed for MSI using the widely adopted combination of mono- and dinucleotide markers (31,33). Ten hMMR cases with a protein loss comprising more than 85% of the tumor were also analyzed. Among breast cancers belonging to the dMMR category, 68 (91%) tumors were microsatellite-stable, as shown in Figure 2 and Table 2

**Table 2.** MMR and microsatellites alterations frequency in dMMR breast cancers*

Characteristic	No. of patients (%)
dMMR patients	75
MLH1	
Retained	28 (37)
Heterogeneous	6 (8)
Loss	41 (55)
MSH2	
Retained	12 (16)
Heterogeneous	8 (11)
Loss	55 (73)
MSH6	
Retained	38 (51)
Heterogeneous	13 (17)
Loss	24 (32)
PMS2	
Retained	51 (68)
Heterogeneous	3 (4)
Loss	21 (28)
No. of MMR markers with IHC loss	
1	34 (45)
2	24 (32)
3	8 (11)
4	9 (12)
Microsatellite instability	
High	1 (1)
Low	6 (8)
Negative	68 (91)

*dMMR = mismatch repair deficient; IHC = immunohistochemistry; MLH1 = mutL homolog 1; MMR = mismatch repair; MSH2 = mutS homolog 2; MSH6 = MSH2 = mutS homolog 6; PMS2 = postmeiotic segregation increased 2.

. None of the hMMR breast cancers showed MSI, akin to pMMR tumors. Furthermore, 6/7 (86%) dMMR tumors (#40, #67, #82, #95, #113, and #148) harbored instability in only one locus and were defined as low-frequency MSI, whereas only 1 tumor (#186) was MSI-H (Figure 2; Table 2; Supplementary Figure 3; Supplementary Table 4, available online). No BAT26- and D5S346-unstable breast cancers were observed (Supplementary Figure 3, available online). The low level of overlap between IHC and MSI analysis (1% if considered MSI-H tumors) suggests that the five markers recommended by the National Cancer Institute may underestimate the actual MMR status in breast cancer.

### The Prognostic and Likely Predictive Role of MMR Deficiency in Breast Cancers

**Figure 3.**
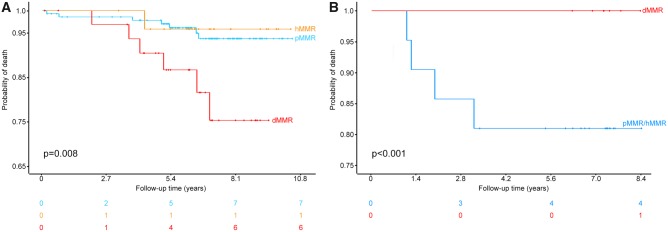
Overall survival of the patients included in the study for selected tumor characteristics on the basis of mismatch repair status. **A**) Probability of death in Luminal B-like breast cancer patients with mismatch repair deficient (dMMR, red) tumors, patients with mismatch repair proficient (pMMR, light blue), and patients with mismatch repair heterogeneous (hMMR, orange); median survival of 77 (range = 0–115) months for the former compared with 84 (0–127) months for the latter two categories. **B**) Probability of death in chemotherapy-treated hormone receptor negative breast cancer patients with mismatch repair proficient (pMMR, light blue), heterogeneous tumors (pMMR/hMMR, dark blue), and mismatch repair deficient tumors (dMMR, red); median survival of 87 (range = 73–123) months for the former compared with 79 (range = 8–113) months for the latter two categories. The curves are built according to the Kaplan-Meier method, *P* values were calculated using two-sided Log-rank tests. Numbers at risk appear below the graphs.

Survival analysis showed different overall survival rates between dMMR and non-dMMR breast cancer patients. Among Luminal B-like carcinomas, patients with pMMR (n = 205) and hMMR (n = 35) tumors lived longer than those with dMMR (n = 44) cancers (*P* = .008), as depicted in Figure 3 A. Specifically, 8 of 240 (3.3%) patients with pMMR or hMMR cancers died of disease after 0–127 (median = 84) months, while 6 (13.6%) of patients with dMMR tumors died after 0–115 (median = 77) months. The prognostic role of MMR deficiency was maintained in chemotherapy-naïve (n = 166, * P* = .003) but not in chemotherapy-treated (n = 118, *P* = .181) patients. Of note, both the risks of death and recurrence were similar among women with pMMR and hMMR breast cancers irrespective of the clinicopathologic and molecular characteristics (*P* > .05). Interestingly, survival analyses of HER2-type and TNBC patients based on MMR system activation showed a completely different scenario (Figure 3B). Taking together the ER-negative patients, whose clinicopathologic features are detailed in Supplementary Table 4 (available online), dMMR tumors (n = 9) showed a better prognosis both in terms of overall and disease-free survival compared with pMMR (n = 33) and hMMR (n = 7) neoplasms (*P* < .001), with 87 months of median survival (range = 73–123 months) for the former compared with 79 months (range = 8–113 months) for the latter two categories (*P* < .001). All of these patients underwent first-line chemotherapy. On the contrary, the MMR status did not affect the prognosis of Luminal A-like tumors. No statistically significant correlations between MMR deficiency and clinicopathologic features were observed. The number of MMR proteins showing IHC loss was not associated with statistically significant increased risks of death and/or recurrence in any subgroup of patients.

### Clinicopathologic Features of MMR-Deficient Breast Cancers

The median age at diagnosis of the 75 patients with dMMR breast cancer was 65 years (range = 31–85 years), higher than in pMMR patients (Table 3

**Table 3.** Clinicopathologic features of dMMR breast cancers compared with pMMR*

	dMMR	hMMR	pMMR
Characteristic	(n = 75)	(n = 55)	(n = 314)
Median age at diagnosis, y	65	60	61
Histological subtype, No. (%)			
NST	59 (79)	48 (87)	237 (76)
Lobular	9 (12)	2 (4)	42 (13)
Other	7 (9)	5 (9)	35 (11)
Histological grade, No. (%)			
G1	6 (8)	5 (9)	35 (11)
G2	32 (43)	21 (38)	137 (44)
G3	37 (49)	29 (53)	142 (45)
T pathologic staging, No. (%)			
pT1	38 (50)	32 (58)	192 (61)
pT2	29 (39)	19 (35)	99 (32)
pT3	2 (3)	1 (2)	7 (2)
pT4	6 (8)	3 (5)	16 (5)
N pathologic staging, No. (n)			
pN0	35 (47)	31 (56)	191 (61)
pN1	25 (33)	10 (18)	78 (25)
pN2	10 (13)	11 (20)	21 (7)
pN3	3 (7)	3 (6)	24 (8)
Hormone receptor positive, No. (%)	65 (87)	49 (89)	277 (88)
Ki67 high, No. (%)	43 (57)	37 (67)	222 (71)
HER2-positive, No. (%)	13 (17)	15 (27)	53 (17)

*dMMR = mismatch repair deficient; ER = estrogen receptor; NST = invasive carcinoma of no special type (ductal); pMMR = mismatch repair proficient (including heterogeneous cases); PR = progesterone receptor.

). Most dMMR breast cancers were invasive ductal carcinomas of no special type (79%) and encompassed 21 (28%) Luminal A-like, 44 (59%) Luminal B-like, 1 (1%) HER2-type, and 9 (12%) TNBCs (Figure 2; Table 3). Thirty-four (45%) dMMR tumors harbored complete loss of expression of one protein, while in 24 (32%) and 8 (11%) cancers the loss involved protein pairs and triplets, respectively (Figure 2; Table 2). Of note, 9 (12%) tumors displayed uniform loss of all four MMR proteins. Taken together, no specific patterns of MMR protein loss were observed across dMMR breast cancers. Forty-one (55%) carcinomas harbored homogeneous loss of MLH1, 55 (73%) of MSH2, 24 (32%) of MSH6, and 21 (28%) PMS2 (Table 2). The latter was the most stable MMR protein in the tumor samples, showing heterogeneous expression in only three cases, as confirmed by the analysis of full sections. The highest level of intra-tumor heterogeneity in MMR protein expression was observed for MSH6.

## Discussion

Our study documents the clinical impact of MMR testing in a large series of breast cancers by means of the most commonly adopted diagnostic tools and criteria. We show that MMR protein loss is a rather common event in breast cancer and shows a remarkable degree of intra-tumor heterogeneity, therefore making the analysis of a small area of the tumor, or a small biopsy, of little clinical value. Our investigation supports the concept that MSI occurs rarely in breast cancer (9) and demonstrates that this condition is restricted to a minority of tumors with MMR protein loss. These data suggest that MMR IHC and MSI analysis should not be considered as interchangeable tests in the diagnostic workup of breast carcinomas. Finally, our observations indicate that the complete loss of at least one of the MMR proteins assessed by IHC is able to identify high-risk Luminal breast cancers that might potentially benefit from pembrolizumab therapy, whereas first-line chemotherapy shows comparatively good results in dMMR TNBCs.

The histology-agnostic approval of the monoclonal antibody pembrolizumab by the US Food and Drug Administration in all dMMR and/or MSI-H tumors (7) opened new avenues for the clinical management of breast cancer patients. This unprecedented decision, however, was based on 149 patients with MSI-H or dMMR cancers enrolled in five single-group clinical trials (8). Most of these patients (84%) had colorectal cancer, whereas only a few of them had breast cancer. Given the current focus on precision immuno-oncology, tumor-specific companion diagnostic tests are warranted (34). Because breast cancer is the most frequent malignant tumor in women (35), the implementation of a reliable MMR diagnostic strategy would be potentially beneficial in identifying patients eligible for immune-checkpoint blockade. For this reason, we decided to apply to breast cancer the widely-employed diagnostic algorithms for MMR status assessment and characterize their clinical consistency.

To our knowledge, this is the first study aiming to define the clinical consequences of intra-tumor heterogeneity in breast cancer MMR testing. Hence, intra-tumor heterogeneity is a major problem in biomarkers assessment for making decisions about treatment, particularly on small tissue samples (eg, core biopsies) (36–38). In this study, we have provided novel evidence that the MMR proteins are heterogeneously expressed in more than 12% of breast cancers, with no preferential distribution inside the tumor. The therapeutic implications of intra-tumor heterogeneity of the MMR phenotype are yet to be ascertained and require dedicated clinical prospective studies. Conversely, the possibility of heterogeneous MLH1, MSH2, MSH6, and PMS2 distribution should be considered while performing MMR testing. Our findings suggest that MMR analysis by IHC in breast cancer requires an extensive sampling of the lesion in surgical resections and should be performed on multiple, possibly all, tumor blocks. The intrinsically low sensitivity of this test in bioptic samples should be taken into account in unresectable advanced tumors and in presurgical samples for neoadjuvant studies.

Massively parallel sequencing studies have demonstrated that approximately 4% of breast cancers harbor MMR alterations in the tumor cells, as confirmed by public genomic data (9,39–42). Our analysis of 444 breast cancers documented a higher proportion of dMMR tumors, where 17% of carcinomas showed complete IHC loss of at least one MMR protein in all neoplastic areas. Interestingly, the condition of MMR deficiency is not related to the histological and/or molecular type. Furthermore, in contrast to endometrial and colorectal cancer, an exceedingly small proportion of dMMR tumors were found to be microsatellite unstable, whereas only one case was MSI-H using the five standard MSI markers. Many seminal works aimed to investigate the usefulness of MSI analysis in breast cancer, with conflicting results. Taken together, more than 300 different microsatellite markers have been tested for MSI assessment in breast cancer, showing marked variations in terms of detection rates (43). Although most of these markers have demonstrated to be largely uninformative, D17S250 has been reported as the most reliable for its positive predictive value (43). In our series, however, only one case showed instability in D17S250, confirming that the choice of microsatellite markers may affect the MSI detection rate. The conflict between the MSI model in colorectal and endometrial cancers and that of breast cancer suggests that not all breast carcinomas harboring defects in DNA repair systems display high levels of genomic instability, as recently reported in homologous recombination repair-deficient breast cancers (44,45). Our study is the first to our knowledge to assess the clinical reliability of this widely adopted diagnostic platform in breast malignancies, and the results provide strong evidence against the interchangeability of IHC and state-of-the-art MSI analysis in these patients. This represents an additional consideration in patients’ selection for immunotherapy. It remains to be determined which patients would benefit more from pembrolizumab administration between IHC-negative/MSI-positive and IHC-negative/MSI-negative breast cancers, requiring further large-scale multicentric studies.

The clinical importance of MMR testing in breast cancers has implications for the prognostic value of IHC, which is unrelated to MSI investigation. Spatial heterogeneity analysis of MMR protein expression revealed similar results in terms of prognosis between hMMR and pMMR breast cancers. These data suggest that the partial loss of the MMR proteins does not represent a detrimental condition for the MMR system in breast cancer, as also reported in endometrial cancer (14). Here, we have demonstrated that the overall survival rates of dMMR and non-dMMR breast cancers are radically different. Interestingly, dMMR Luminal B-like patients showed a worse prognosis compared with both pMMR and hMMR, particularly among chemotherapy-naïve patients. These data suggest that this specific molecular subgroup of patients could be an appropriate target for immune-checkpoint inhibition. Clinical trials designed to compare the performance of first-line immunotherapy (alone or in combination) with the traditional therapeutic approaches in ER-positive breast cancers would be needed. In contrast to what we have observed in Luminal B-like tumors, dMMR chemotherapy-treated ER-negative breast cancers had an excellent outcome both in terms of disease-free survival and overall survival. These observations provide circumstantial evidence to suggest that phenomena of synthetic lethality (4) might improve the efficacy of chemotherapy in these tumors. Definition of the clinical rationale of pembrolizumab administration in these patients and whether MMR testing by IHC can be employed as a biomarker of prognosis and chemotherapy efficacy require validation in independent cohorts of breast cancer patients.

This study has some limitations. First, we did not analyze the germ lines of our patients to identify and subsequently exclude syndromic breast cancers. However, the analysis of *MLH1* promoter methylation coupled with clinical and family information has been shown to represent a rational testing surrogate for Lynch syndrome, both in the clinic and in breast cancer translational research studies (9,11,46). Furthermore, we are aware that the use of TMAs might underestimate the intra-tumor heterogeneity. To reduce this possible drawback, we have analyzed full sections of all dMMR tumors. In addition, next-generation sequencing studies have recently provided evidence to suggest that dMMR tumors exhibit a hypermutator phenotype, but we did not perform these analyses. Despite these limitations, we aimed to define for the first time in breast cancer the reliability of the MMR testing methods currently employed in clinical practice (ie, IHC and MSI analysis). Further clinical trials coupled with massively parallel sequencing studies are needed to assess the role of mutational burden as a predictive biomarker for immune checkpoint blockade. Finally, given the relatively small number of tumors analyzed, caution should be exercised in the interpretation of the clinical impact of our observations. Hence, this study should be considered hypothesis generating. Further investigations including larger cohorts of patients are warranted to elucidate the clinical role of MMR testing in breast cancer.

Our results indicate that the IHC analysis of multiple areas of the tumor, regardless of microsatellite status, has a prognostic and likely predictive role in both Luminal B-like and ER-negative breast cancers. Further development of reliable testing for MMR in breast cancer is required to fully understand the prognostic and predictive role of MMR status, to further select those patients who may benefit from systemic therapy, and to facilitate the rational testing and use of immunotherapeutics in breast cancer.

## Funding

This study was supported by Fondazione IRCCS Ca’ Granda - Ospedale Maggiore Policlinico (Stefano Ferrero Ricerca Corrente 2017).

## Notes

Affiliations of authors: Division of Pathology, Fondazione IRCCS Ca’ Granda, Ospedale Maggiore Policlinico, Milan, Italy (NF, GL, CC, CP, PC, GE, GG, AF, AM, VV, MM, SF, SB); Department of Biomedical, Surgical, and Dental Sciences (NF, AF, SF) and Department of Pathophysiology & Transplantation (CP, PC, VV, MM), Università degli Studi di Milano, Milan, Italy; Division of Medical Oncology (DG, AM) and Division of Breast Surgery (LD, CB), Fondazione IRCCS Ca’ Granda - Ospedale Maggiore Policlinico, Milan, Italy.

## Supplementary Material

Supplementary Data
