# Supplementary material for: Mismatch Repair Protein Loss as a Prognostic and Predictive Biomarker in Breast Cancers Regardless of Microsatellite Instability
Source: JNCI Cancer Spectr. 2018 Dec 13;2(4):pky056. doi: 10.1093/jncics/pky056 (PMC6649738; doi:10.1093/jncics/pky056)
Supplement: Supplementary Data [file pky056_supp.docx]

**Mismatch repair proteins loss is a prognostic and predictive biomarker in non-familial breast cancers regardless of microsatellite instability**

**SUPPLEMENTARY MATERIALS**

| **Marker** | **Clone** | **Dilution** | **Company/technology** | **Antigen retrieval** | **Scoring** |
| --- | --- | --- | --- | --- | --- |
| ER | EP1 | RTU | Dako Omins | EnVision FLEX, High pH, 20' | ASCO/CAP and St Gallen guidelines; positive if ≥1% of tumor cell nuclei are immunoreactive, high if >20% of tumor cell nuclei are immunoreactive |
| PR | PgR 636 | 1:100 | Dako Omnis | EnVision FLEX, High pH, 30’ | ASCO/CAP and St Gallen guidelines; positive if ≥1% of tumor cell nuclei are immunoreactive, high if >20% of tumor cell nuclei are immunoreactive |
| Ki67 | MIB1 | RTU | Dako Omnis | EnVision FLEX, High pH, 30’ | ASCO/CAP and St Gallen guidelines; high if >20% of tumor cell nuclei are immunoreactive |
| HER2 | Polyclonal | 1:400 | Dako Omnis | EnVision FLEX, Low pH, 30' | ASCO/CAP guidelines; 3+ if uniform intense membrane staining circumferential membrane staining that is complete and intense, 2+ if circumferential membrane staining that is incomplete and/or weak/moderate and within >10% of the invasive tumor cells or complete and circumferential membrane staining that is intense and within ≤10% of the invasive tumor cells, negative for other staining patterns |
| MLH1 | ES05 | 1:50 | Ventana Benchmark Ultra | CC1, 68’ | Negative if complete loss of nuclear staining within all tumor cells |
| MSH2 | FE11 | 1:50 | Ventana Benchmark Ultra | CC1, 68’ | Negative if complete loss of nuclear staining within all tumor cells |
| MSH6 | EP49 | 1:100 | Ventana Benchmark Ultra | CC1, 68’ | Negative if complete loss of nuclear staining within all tumor cells |
| PMS2 | EP51 | 1:50 | Ventana Benchmark Ultra | CC1, 68’ | Negative if complete loss of nuclear staining within all tumor cells |
| **Supplementary Table S1. List of antibodies, clones, dilutions, antigen retrieval methods, and scoring systems adopted for immunohistochemical analyses.** ER, estrogen receptor alpha; PR, progesterone receptor; MLH1, ; MSH2, ; MSH6, ; PMS2, ; RTU, ready to use. | | | | | |

| **Microsatellite marker** | **Location** | **Forward primer** | **Reverse primer** |
| --- | --- | --- | --- |
| BAT25 | *KIT* (4q12) | 5’-[6FAM]-TCGCCTCCAAGAATGTAAGT-3’ | 5’-TCTGCATTTTAACTATGGCTC-3’ |
| BAT26 | *MSH2* (2p21-p16.3) | 5’-[6FAM]-TGACTACTTTTGACTTCAGCC-3’ | 5’-AACCATTCAACATTTTTAACCC-3’ |
| D2S123 | *MSH6* (2p16.3) | 5’-[6FAM]-AAACAGGATGCCTGCCTTTA-3’ | 5’-GGACTTTCCACCTATGGGAC-3’ |
| D5S346 | *APC* (5q22.2) | 5’-[6FAM]-ACTCACTCTAGTGATAAATCGGG-3’ | 5’-AGCAGATAAGACAGTATTACTAGTT-3’ |
| D17S250 | *BRCA1* (17q21.31) | 5’-[6FAM]-GGAAGAATCAAATAGACAAT-3’ | 5’-GCTGGCCATATATATATTTAAACC-3’ |
| **Supplementary Table S2**. **Primer sequences for the detection of microsatellite instability in 130 dMMR and hMMR breast cancers.** | | | |

|  | **Total (%)** | | **Luminal A** *n=108* | **Luminal B (HER2-)**  *n=211* | | **Luminal B (HER2+)**  *n=73* | | | **HER2-type**  *n=8* | | **TNBC**  *n=44* |
| --- | --- | --- | --- | --- | --- | --- | --- | --- | --- | --- | --- |
| pMLH1 | 371 (84) | 89 (82) | | | 182 (86) | | 57 (78) | 7 (88) | | 36 (82) | |
| dMLH1 | 41 (9) | 10 (9) | | | 16 (8)  | | 9 (12)  | 0 | | 6 (14)  | |
| hMLH1 | 32 (7) | 9 (8)  | | | 13 (6)  | | 7 (10)  | 1 (12)  | | 2 (4)  | |
| pMSH2 | 347 (78) | 82 (76) | | | 170 (81) | | 54 (74) | 5 (63) | | 36 (82) | |
| dMSH2 | 55 (12)  | 15 (14)  | | | 23 (11)  | | 10 (14)  | 1 (12)  | | 6 (14)  | |
| hMSH2 | 42 (10)  | 11 (10)  | | | 18 (8)  | | 9 (12)  | 2 (25)  | | 2 (4)  | |
| pMSH6 | 396 (90) | 93 (86) | | | 190 (90) | | 63 (86) | 8 (100) | | 42 (96) | |
| dMSH6 | 24 (10)  | 7 (6)  | | | 9 (4)  | | 6 (8)  | 0 | | 2 (4)  | |
| hMSH6 | 24 (10)  | 8 (8)  | | | 12 (6)  | | 4 (6)  | 0 | | 0 | |
| pPMS2 | 416 (94) | 104 (96) | | | 201 (95) | | 66 (90) | 8 (100) | | 37 (84) | |
| dPMS2 | 21 (5) | 2 (2)  | | | 7 (3)  | | 6 (8)  | 0 | | 6 (14)  | |
| hPMS2 | 7 (1)  | 2 (2)  | | | 3 (2)  | | 1 (2)  | 0 | | 1 (2)  | |
| **Supplementary Table S3. Mismatch repair protein status assessed by immunohistochemistry according to the surrogate definitions of intrinsic molecular subtypes.** Black bars depict the percentage of tumors among those showing homogeneous and heterogeneous patterns of protein loss. Prefixes: p, proficient; d, deficient; h, heterogeneous. | | | | | | | | | | | |

|  | **Luminal A-like***  **n=108** | **Luminal B-like**^#^  **n=284** | **HER2-type**^∞^  **n=8** | **TNBC**^§^  **n=44** |
| --- | --- | --- | --- | --- |
| Age at diagnosis, n (%) |  |  |  |  |
| ≥55 years | 80 (74) | 203 (72) | 6 (75) | 22 (50) |
| <55 years | 28 (26) | 81 (28) | 2 (25) | 22 (50) |
| Histological subtype, n (%) |  |  |  |  |
| Invasive carcinoma, NST | 62 (57) | 240 (85) | 6 (75) | 36 (82) |
| Lobular | 29 (27) | 24 (9) | 0 | 0 |
| Other | 17 (16) | 20 (6) | 2 (25) | 8 (18) |
| Histological grade, n (%) |  |  |  |  |
| 1 | 20 (19) | 24 (9) | 0 | 2 (5) |
| 2 | 77 (71) | 108 (38) | 1 (13) | 4 (9) |
| 3 | 11 (10) | 152 (53) | 7 (97) | 38 (86) |
| Hormone receptor status, n (%) |  |  |  |  |
| Positive | 108 (100) | 284 (100) | 0 | 0 |
| Negative | 0 | 0 | 8 (100) | 44 (100) |
| HER2 status, n (%) |  |  |  |  |
| Positive | 0 | 211 (74) | 8 (100) | 0 |
| Negative | 108 (100) | 73 (26) | 0 | 44 (100) |
| Ki67 status, n (%) |  |  |  |  |
| High | 0 | 253 (89) | 7 (97) | 42 (95) |
| Low | 108 (100) | 31 (11) | 1 (13) | 2 (5) |
| Stage, n (%) |  |  |  |  |
| I | 43 (40) | 118 (42) | 2 (25) | 17 (39) |
| II | 54 (50) | 99 (35) | 2 (25) | 18 (41) |
| III-IV | 11 (10) | 67 (23) | 4 (50) | 9 (20) |
| Mismatch repair status, n (%) |  |  |  |  |
| Proficient | 73 (68) | 205 (72) | 5 (63) | 31 (70) |
| Deficient | 21 (19) | 44 (15) | 1 (12) | 9 (21) |
| Heterogeneous | 14 (13) | 35 (13) | 2 (25) | 4 (9) |
| Microsatellite status, n (%) |  |  |  |  |
| Stable | 108 (100) | 278 (98) | 8 (100) | 43 (98) |
| High instability | 0 | 0 | 0 | 1 (2) |
| Low instability | 0 | 6 (2) | 0 | 0 |
| **Supplementary Table S4.** **Clinicopathologic features of the intrinsic molecular subtypes of breast cancer included in the study.** NST, no special type; *ER+/PR+/Ki67 low; ^#^ER+/Ki67 high or ER+/PR-; ^∞^ER-/PR-/HER2+; ^§^ER-/PR-/HER2-. | | | | |

| 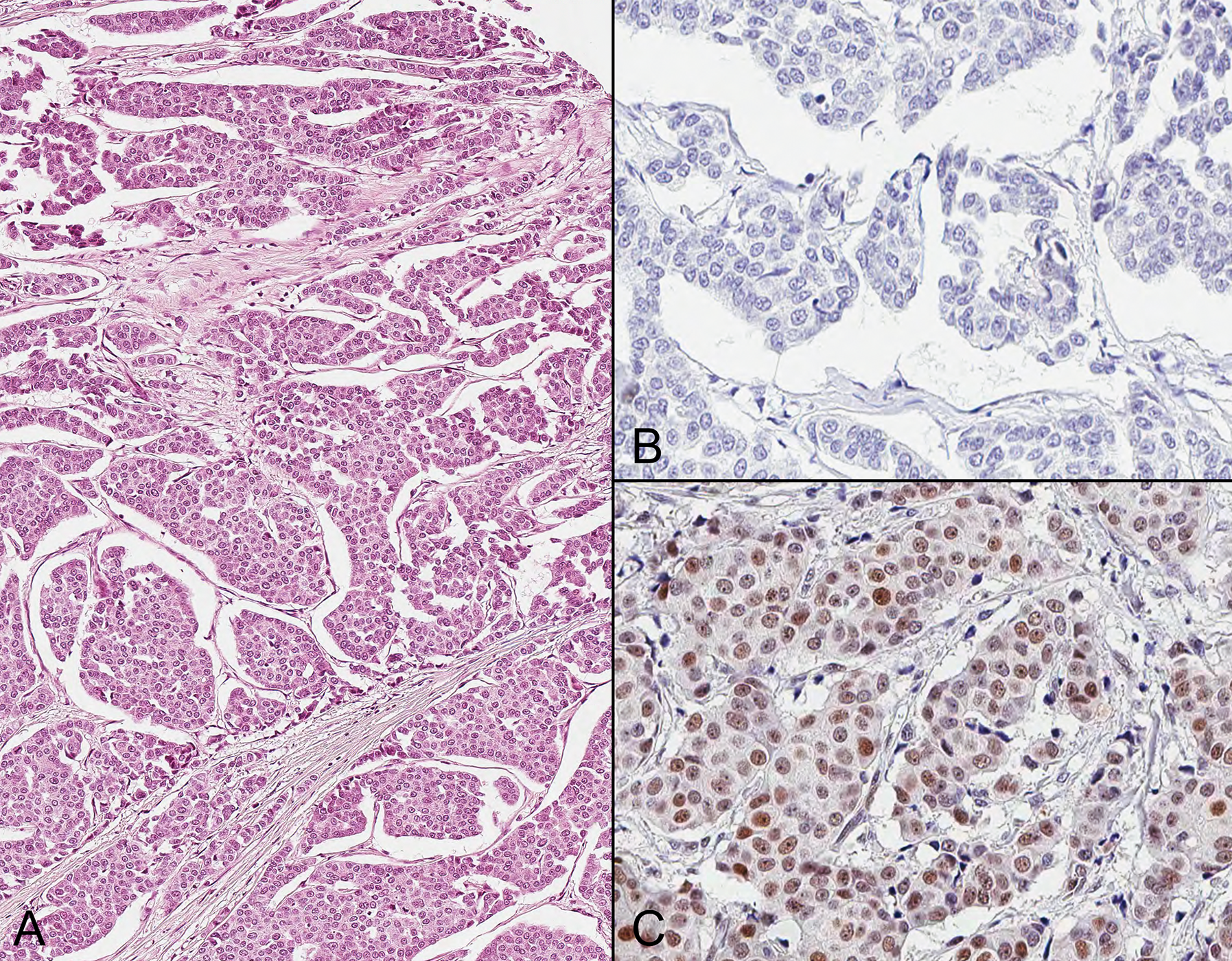 |
| --- |
| **Supplementary Figure S1. Representative micrographs of a mismatch repair deficient breast cancer with heterogeneous immunohistochemical loss of MSH1.** Case 358 was a moderately differentiated (G2) invasive carcinoma of no special type (A, original magnification 100x). This tumor showed areas of spatially heterogeneous expression of the protein, where a predominant MLH1-negative tumor population (B) coexisted with a minor area of MLH1-positive neoplastic cells (C), in the presence of homogeneous morphology. |

| 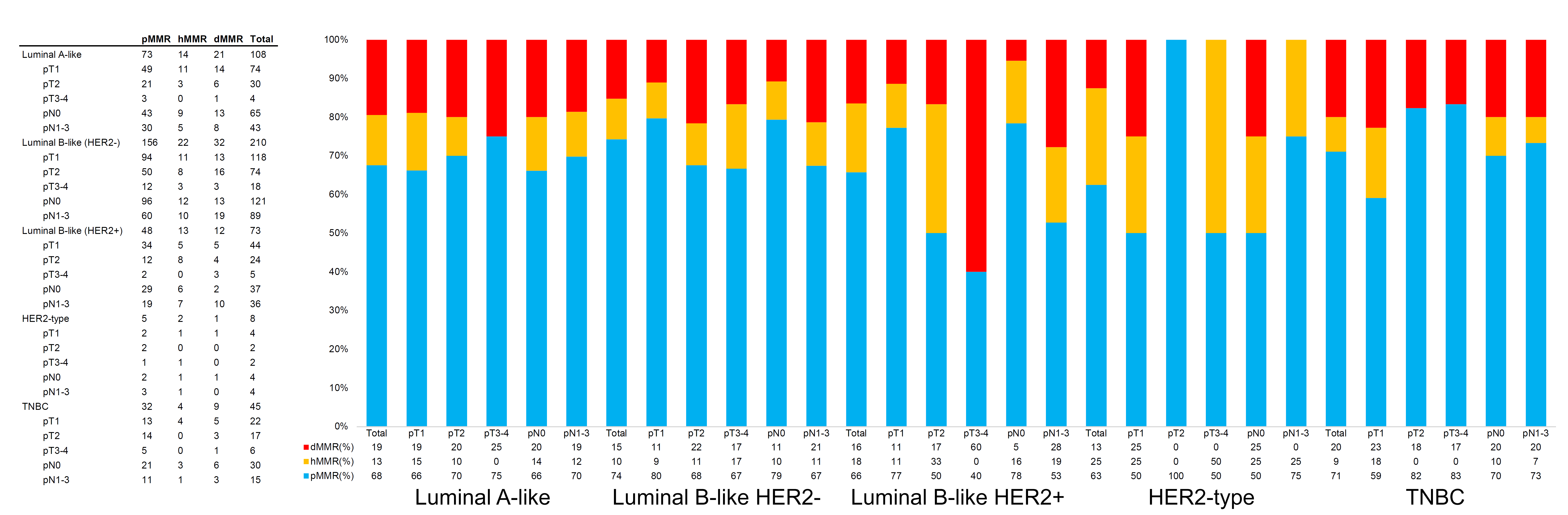 |
| --- |
| **Supplementary Figure S2. TNM breakdown of the intrinsic molecular subtypes with the corresponding MMR status.** dMMR, mismatch repair deficient; hMMR, mismatch repair heterogeneous; pMMR mismatch repair proficient. |

| 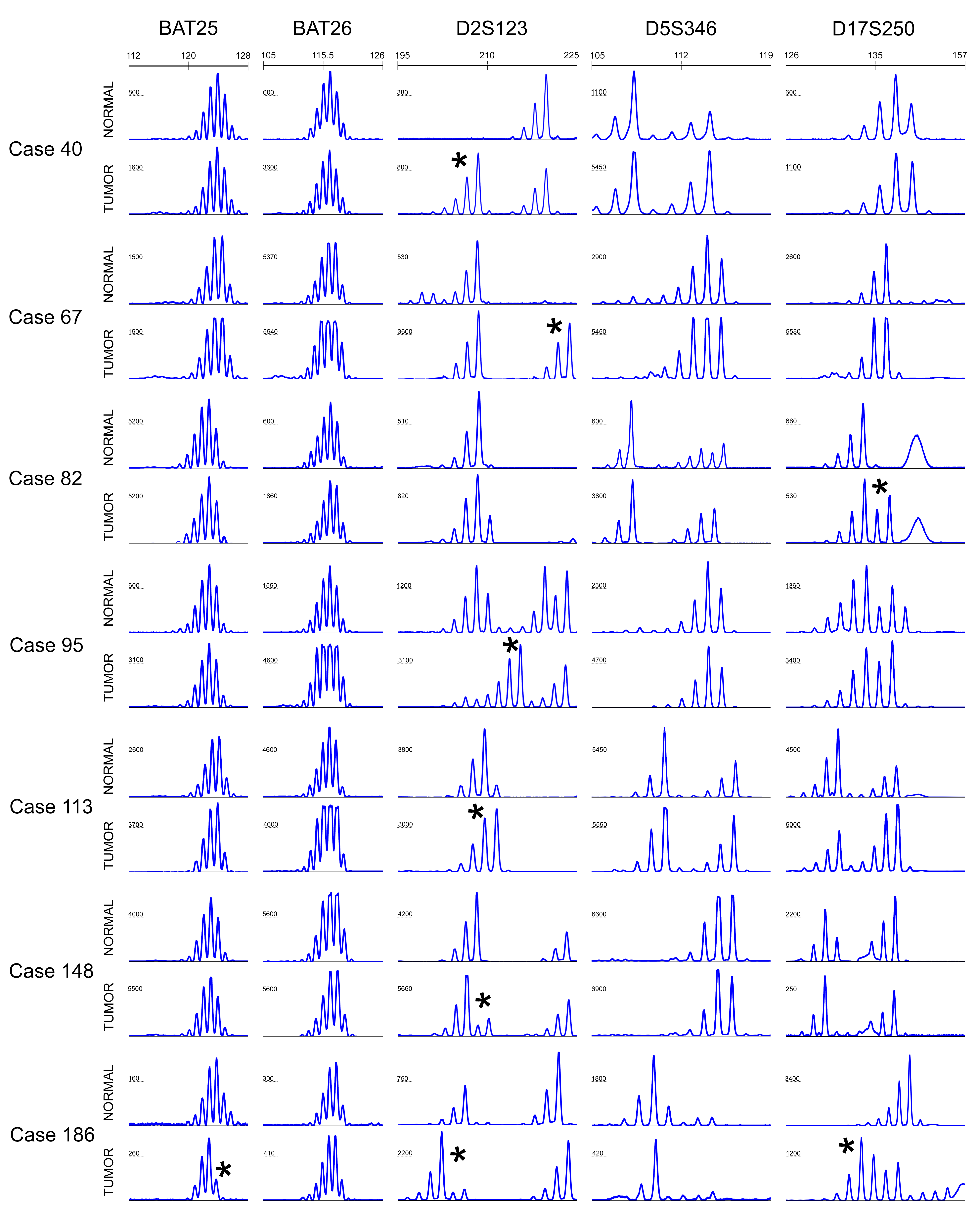 |
| --- |
| **Supplementary Figure S3. Microsatellite traces of 7 breast carcinomas showing mismatch repair protein loss and microsatellite instability.** For each case, the electropherograms of tumor and matched normal tissue are represented according to the 5 microsatellite markers BAT25, BAT26, S2S123, D5S345, and D17S250. The unstable regions are highlighted by stars. |
